# Synthesis of Lithium Iron Phosphate Materials via an All-in-One Integrated Liquid Phase Method

**DOI:** 10.3390/molecules31091419

**Published:** 2026-04-25

**Authors:** Shixiang Sun, Bo Liao, Xiaotao Wang, Han Wu, Jinyu Tan, Jingwen Cui, Yingqun Li, Wei Li, Yidan Zhang, Siqin Zhao, Yan Cao, Chao Huang

**Affiliations:** 1 College of Physics and Electronic Information, Inner Mongolia Normal University, Hohhot 010022, China; 2 Inner Mongolia Key Laboratory of Applied Condensed Matter Physics, Hohhot 010022, China; 3 Inner Mongolia Engineering Research Center for Rare Earth Functional and New Energy Storage Materials, Hohhot 010022, China; 4 China Tower Corporation Inner Mongolia Autonomous Region Branch, Hohhot 010050, China; 5 Inner Mongolia Shengfan Technology Co., Ltd., Hohhot 010022, China

**Keywords:** lithium-ion battery, lithium iron phosphate, integration of all elements, lithium-ion mobility

## Abstract

Lithium iron phosphate (LiFePO_4_) (LFP) has emerged as the most popular cathode material in the current lithium battery market because of its stable charge–discharge cycle performance, low cost, and high safety. Moreover, this material does not require scarce resources such as nickel and cobalt, which alleviates supply chain conflicts and reduces the environmental and health impacts associated with Ni and Co. In this study, a cost-effective preparation method is implemented to synthesize a series of all-element integrated LiFePO_4_ precursors using precursor solutions with varying concentrations of oxalic acid. The final LFP materials are subsequently obtained through a one-step heat treatment. To evaluate the advantages of this method, we compare the structural and electrochemical properties of the obtained LFP materials with those synthesized via the traditional solid-phase method. The experimental results reveal that the LFP material synthesized using an oxalic acid solution with a concentration of 0.125 mol L^−1^ exhibits optimal performance. This material has a grain size in the range of 300–500 nm, which is smaller and more uniform than those of the other samples. This initial specific discharge capacity of the designed LFP is 150.3 mAh·g^−1^, with an initial coulombic efficiency of 88%. Notably, the material maintains a high capacity of 98 mAh·g^−1^ even at −20 °C and achieves a discharge capacity of 98.7 mAh·g^−1^ at a high discharge rate of 5 C. The lithium-ion diffusion coefficient was determined to be 7.1 × 10^−12^ cm^2^ s^−1^, which is approximately 2.5 times greater than that of the material synthesized via the solid-phase ball-milling method. These results highlight the significant improvements in both the structural and electrochemical properties of LFP materials synthesized through this novel liquid-phase method.

## 1. Introduction

Lithium-ion batteries have been widely adopted because of their high energy density, stable cycle performance, and environmental friendliness. After more than half a century of development, related technical methods have become highly mature and extensively applied in electric vehicles and portable electronic devices [1,2]. At present, the cathode materials of commercial lithium-ion batteries primarily include lithium cobalt oxide, ternary nickel–cobalt–manganese lithium material, and lithium iron phosphate (LFP). Compared with other lithium-ion battery cathode materials, LFP cathode materials have advantages such as a wide range of available raw materials, low cost, high safety and low environmental impact. These attributes are particularly beneficial for reducing battery costs and promoting the large-scale development of lithium-ion batteries [3,4,5]. The LiFePO_4_ crystal has an olivine structure, with a theoretical specific capacity of 170 mAh·g^−1^ and a discharge platform exceeding 3.4 V. When fully charged, the olivine structure of LFP exhibits excellent stability and a high reversible specific capacity [6].

Three main synthesis methods are employed for LFP materials: the high-temperature solid-phase method, the carbon-thermal reduction method, and the hydrothermal method [7]. Moreover, other widely used methods include coprecipitation, sol–gel, and microwave-assisted synthesis. For industrial-scale production, both the high-temperature solid-phase method and carbon-thermal reduction method are relatively simple solid-phase methods. However, these methods are characterized by long mixing times and high energy consumption [8,9]. More critically, the particle morphology of LFP materials significantly influences the lithium-ion conductivity. Although microwave synthesis is fast and has a short cycle length, a reasonable response time also needs to be controlled [10]. Samples produced via the solid-phase method exhibit insufficient uniformity in terms of particle size and morphology, leading to poor ionic conductivity and reduced practicality for use in batteries [11,12]. In contrast, LFP materials synthesized via hydrothermal and sol–gel methods tend to have smaller powder sizes and more uniform particles. However, the former requires equipment capable of withstanding high temperatures and pressures, whereas the latter has high production costs. Scaling up these methods for actual production necessitates addressing issues related to cost and safety [13,14]. Unlike the hydrothermal method, the coprecipitation method requires a lower temperature and does not require the manufacturing of special equipment in high-temperature and high-pressure environments. This method has the advantages of low energy consumption and low equipment cost [15,16]. Given these considerations, research on the synthesis of LFP materials has focused mainly on two major directions: (i) cost optimization, which involves improving the preparation process to reduce the cost of material synthesis; and (ii) improved ionic conductivity, which involves the use of ion doping and surface coating techniques to solve the problem of insufficient conductivity. In addition, potential environmental impacts during the synthesis process should also be considered, such as the toxicity of any dopants used. In this regard, compared with other samples, the lithium iron phosphate material synthesized by the all-in-one integrated liquid phase method has a smaller volume and is more uniform. Moreover, this material can maintain good performance at high rates and low temperatures, making its synthesis cost-effective.

In this study, FeC_2_O_4_·2H_2_O, LiOH·H_2_O, and NH_4_H_2_PO_4_ were utilized as raw materials to form a series of LFP precursors in oxalic acid solutions of varying concentrations at room temperature and ambient pressure. A series of LFP cathode materials was subsequently obtained through one-step calcination. By comparing the charge–discharge performances of the synthesized cathode materials with that of a cathode material synthesized by a typical solid-phase method, the optimal oxalic acid concentration for synthesizing LFP precursors was determined. Furthermore, the structural properties of the samples obtained through different synthesis methods were analyzed to explore their interrelationships. The aims of these investigations are to optimize the synthesis process and enhance the electrochemical performance of LFP materials, paving the way for their broader application in next-generation lithium-ion batteries.

## 2. Results and Discussion

### 2.1. XRD Analysis

**Figure 1:**
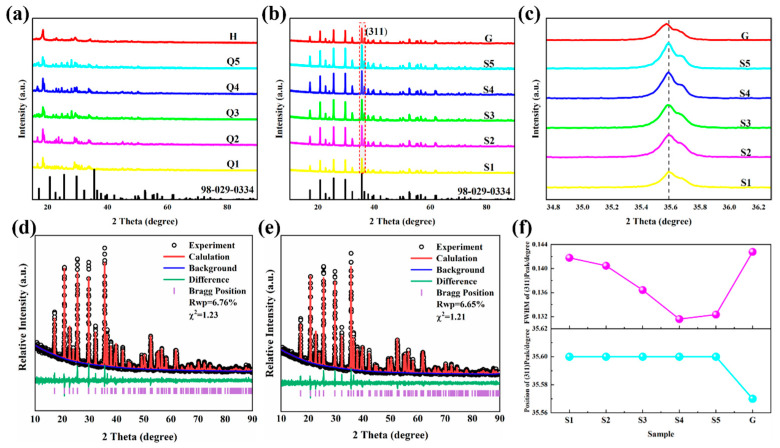
Structural characterization. (**a**) XRD pattern of the precursor. (**b**,**c**) XRD patterns of S1–S5 and G. (**d**,**e**) Observed/calculated XRD patterns of S4 and G. (**f**) Half-high width and position of the (311) peak for different samples.

The XRD patterns of different precursor samples are shown in Figure 1a. These patterns reveal that precursors Q1–Q5 synthesized via the all-element liquid-phase method exhibit more diffraction peaks, with some of them beginning to align with the standard LiFePO_4_ card (98-029-0334) [17]. This observation indicates that, in a liquid-phase environment, the Li, Fe, P, and O in solution started to form specific chemical bonds, demonstrating the ordering and emergence of crystalline characteristics in the precursor. In contrast, the XRD pattern of precursor H after solid-phase ball milling shows no significant additional diffraction peaks, suggesting that the ball milling process results in only a mechanical mixture and does not involve chemical bond breaking, rearrangement, or chemical changes. The XRD patterns of different LiFePO_4_ samples are shown in Figure 1b. The results demonstrate that samples S1–S5 synthesized via the all-element liquid-phase method and sample G synthesized via the traditional ball-milling solid-phase method all exhibit sharp diffraction peaks without obvious stray peaks. Furthermore, all the characteristic peaks fully match the standard PDF card (98-029-0334), confirming that these samples belong to the LFP Pnma space group with an olivine-type structure [18,19]. To further compare the differences among the samples, the (311) crystal surface diffraction peaks were enlarged and analyzed, as shown in Figure 1c. The analysis reveals that the peak position of sample G synthesized via the solid-phase method shifts to a slightly smaller angle than the peaks of the samples prepared by the all-element liquid-phase method, and its diffraction peak sharpness is significantly lower than those of the other samples.

For samples S1–S5 synthesized via the all-element liquid-phase method, the diffraction intensities and sharpness vary considerably depending on the concentration of oxalic acid in the precursor solution. To further investigate the effects of different synthesis methods on the crystalline properties of the materials, we performed Rietveld refinement of samples S4 and G using GSAS–II software. The refinement results, which are shown in Figure 1d,e, demonstrate that the Rwp values for both samples are less than 10%, indicating reliable refinement outcomes. As detailed in Table 1, the cell parameters a, b, and c of sample S4 are smaller than those of sample G synthesized via the solid-phase method. On the basis of the existing diffusion theory for LiFePO_4_ materials, an increase in the cell parameters b and c leads to longer Li^+^ diffusion paths within the unit cell, which is unfavorable for Li^+^ migration. This suggests that the Li^+^ diffusion rate in sample G is lower than that in sample S4.

The half-peak widths of the (311) diffraction peaks for the six samples are shown in Figure 1f, with the peak positions plotted accordingly. The results indicate that sample G synthesized via the solid-phase method has the largest half-peak width. For the samples synthesized via the all-element liquid-phase method, the half-peak width decreases progressively with increasing oxalic acid concentration, reaching its minimum value for sample S4 (oxalic acid concentration of 0.125 mol L^−1^). However, further increases in the oxalic acid concentration lead to an increase in the half-peak width. Additionally, although the peak positions of the samples synthesized via the all-element liquid-phase method remain consistent, the peak position of sample G synthesized via the solid-phase method shifts slightly to a smaller angle. This observation, which is consistent with fundamental crystallographic principles, suggests that sample G has a relatively large crystal plane spacing, further supporting the refinement results.

X-ray diffraction (XRD) analysis confirms that both the all-element liquid-phase method and the conventional solid-state method can produce phase-pure LiFePO_4_ (LFP) materials. The sharpness of the XRD peaks indicates that the crystallinity of LFP synthesized via the liquid-phase method is slightly superior to that of the LFP synthesized via the solid-state method. Notably, the crystallization quality of the liquid-phase synthesized LFP is likely influenced by the oxalate concentration in the precursor. This study demonstrates that the LFP material exhibits optimal crystallinity when the oxalate concentration is optimized at 0.125 mol L^−1^.

### 2.2. Surface Morphology and Composition Analysis

**Figure 2:**
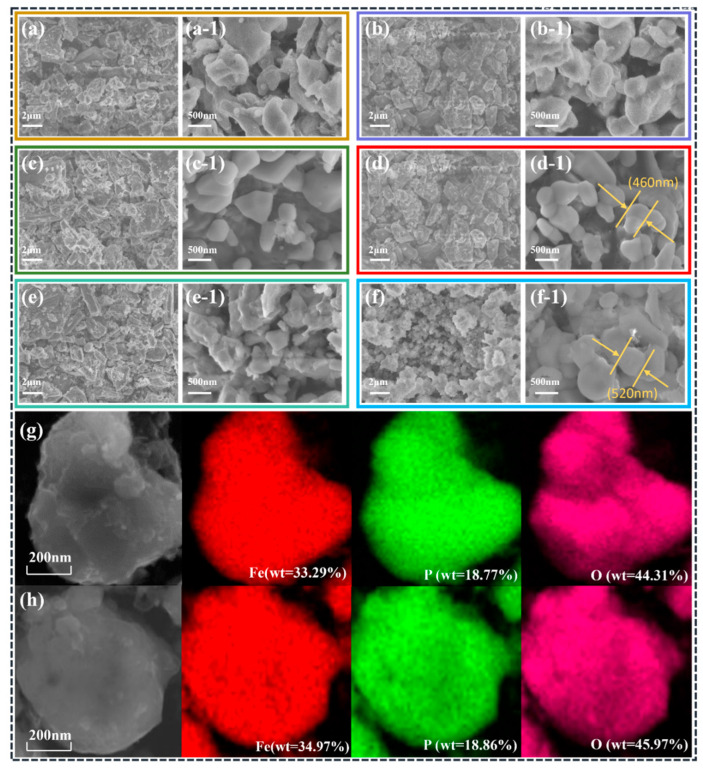
(**a**–**f**) Surface morphology of precursors Q1–Q5 and H, (**a-1**–**f-1**) surface morphology of samples S1–S5 and G, and (**g**,**h**) EDS results.

Scanning electron microscope (SEM) images of the precursors and final products for different samples are shown in Figure 2. The precursors Q1–Q5 synthesized via the all-element integrated liquid-phase method, which exhibit an irregular and dense blocky morphology, are shown in Figure 2a–e, respectively. This indicates that the atoms in the raw materials underwent rearrangement under liquid-phase conditions, resulting in the formation of initial LFP precursors. In contrast, the precursor synthesized by the ball-milling solid-phase method, as shown in Figure 2f, is a homogeneous mixture obtained through high-energy ball milling. The SEM image reveals fine and loose particles, with no evidence of LFP grain formation or atomic rearrangement.

The morphologies of the final LFP samples obtained from different precursors after the same sintering treatment are shown in Figure 2(a-1–f-1), respectively. From a morphological perspective, both methods successfully prepared LFP cathode materials after the same high-temperature treatment. The microscopic morphology of all the samples is typical of LFP materials. Overall, the microscopic morphologies of the LFP samples synthesized by the two methods are largely similar and are all composed of irregular spherical particles stacked together. The primary difference lies in the regularity and uniformity of the spherical particles. The sample synthesized using the oxalic acid precursor has more regular and uniform particle sizes, with a particle size of approximately 300–500 nm. In comparison, the LFP sample synthesized by the ball-milling method has larger particles (approximately 500–700 nm) and a less homogeneous particle size distribution.

With respect to LFP materials, particle size significantly affects ion mobility [20]. On the basis of the SEM results, the ion mobility of samples S1–S5 (synthesized using the oxalic acid precursor) is predicted to be superior to that of sample G (synthesized via the solid-phase ball-milling method). This aligns with the advantages of the liquid-phase method over the solid-phase method, particularly in terms of achieving a more homogeneous particle size and a more centralized particle size distribution. Additionally, as shown in Figure 2g,h, energy dispersive X-ray spectroscopy (EDS) was performed on samples S4 and G. The results reveal that all the elements are successfully distributed on the surface of the samples, indicating that the prepared materials are homogeneous and free of compositional segregation. Quantitative analysis of the surface layers further compared the elemental compositions of S4 and G. The proportions of each element in S4 were slightly lower than those in G.

To more accurately investigate the compositional differences between the two samples, ICP–OES analysis was conducted, and the results are summarized in Table 2. The content of each element in the materials synthesized by the all-element integrated liquid-phase method is slightly lower than that in the materials synthesized by the solid-phase method. This suggests that a certain loss of composition occurs during the precursor synthesis process, which may influence the electrochemical properties of the materials.

**Figure 3:**
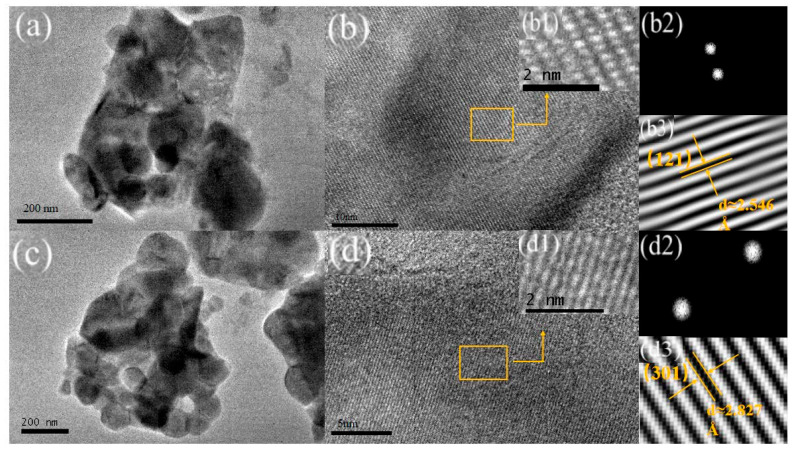
TEM images of S4 (**a**,**b**) and G (**c**,**d**), (**b1**–**b3**) and (**d1**–**d3**) are the results of the FFT patterns in S4 and G, respectively.

**Figure 4:**
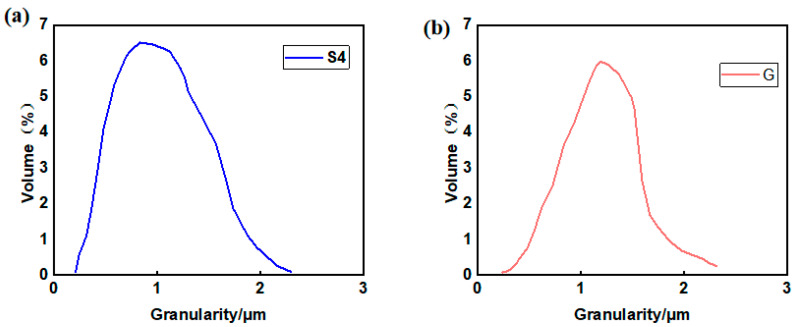
The particle size distribution of sample S4 (**a**) and G (**b**).

To further investigate the effects of different synthesis methods on the morphology and crystal structure of LFP materials, samples S4 and G were examined using transmission electron microscopy (TEM), and the results are presented in Figure 3. The TEM images in Figure 3a,c reveal that both materials exhibit uniform and consistent spherical structures. Notably, by comparing the particle sizes of the two materials in Figure 4a,b, the particle size of sample S4 is mostly clustered around 900 nm, which is significantly smaller than that of sample G 1100 nm. This is consistent with the results of the scanning electron microscope (SEM) analysis. The reduced particle size, particularly the smaller radius of S4, is beneficial for enhancing ion mobility in the material.

High-magnification TEM images of the two LFP materials are shown in Figure 3b,d. Both images display uniformly ordered lattice stripes, which are indicative of well-defined crystalline structures. The high-magnification TEM images were processed using Fourier transform analysis via Digital Micrograph software GMS 3.31, and the results are illustrated as b1–b3 (S4) and d1–d3 (G). The analysis reveals that the interplanar crystal spacings at the corresponding positions are 2.546 Å and 2.827 Å, which correspond to the (121) and (301) crystal planes of the LFP material, respectively. These findings suggest that both synthesis methods yield materials with stable morphologies and well-crystallized structures. In addition, these observations align closely with the XRD results presented in Figure 1, further confirming the structural consistency of the materials.

### 2.3. Thermal Analysis of the Precursors

**Figure 5:**
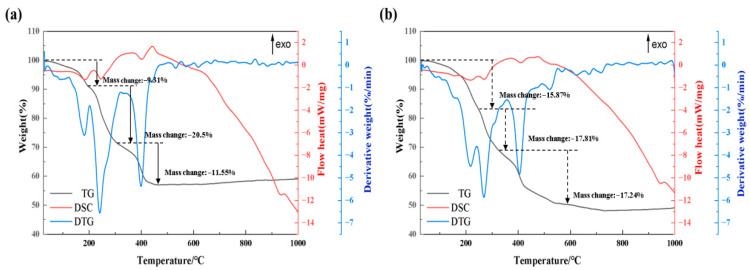
TG–DSC curves of samples Q4 (**a**) and H (**b**).

To investigate the physicochemical differences between the precursors obtained via distinct synthesis methods, we performed simultaneous thermogravimetric and differential scanning calorimetry (TG–DSC) analyses on precursors Q4 and H, and the results are presented in Figure 5. The TG–DSC curve of precursor H is shown in Figure 5a, whereas that of precursor Q4 is shown in Figure 5b. The DSC curves indicate that the general thermal behavior of both precursors follows a similar trend, with no distinct sharp endothermic or exothermic peaks observed. This finding indicates that the thermal effects of the two precursors are not concentrated and that their crystallographic properties are not particularly pronounced.

The TG curves reveal that the mass loss of both precursors can be divided into three distinct stages. The first stage occurs between 25 and 200 °C, where the mass loss primarily corresponds to the removal of physisorbed water from the precursors. Notably, compared with precursor H, precursor Q4 retains a greater amount of surface water because of insufficient drying time, which is obtained in a liquid-phase environment, leading to greater mass loss in this stage. The second stage spans 200–300 °C, during which the mass loss is greater than that in the first stage and corresponds to the removal of crystallization water from the precursors. The mass loss percentages for precursors H and Q4 in this stage are 20.5% and 17.8%, respectively. The lower mass loss of Q4 than that of H can be attributed to the fact that Q4, formed in an oxalic acid solution under controlled reaction conditions, has already undergone partial removal of crystallization water during precursor formation. In contrast, H, a homogeneous mixture obtained via ball milling, retains a higher proportion of crystallization water, resulting in greater mass loss during this stage. The third stage occurs between 300 and 1000 °C and corresponds to the thermal decomposition of the precursor material. The TG–DSC curves reveal that the synthesis methods have a minimal influence on the thermal decomposition process, indicating that the intrinsic thermal reactivity of the materials is largely consistent regardless of the precursor preparation method.

TG–DSC analysis demonstrated that the different synthesis methods have negligible effects on the overall thermal reaction behavior of the materials, suggesting that the physicochemical properties of the precursors are not significantly altered in terms of their thermal stability or decomposition pathways.

### 2.4. XPS Elemental Valence Analysis

**Figure 6:**
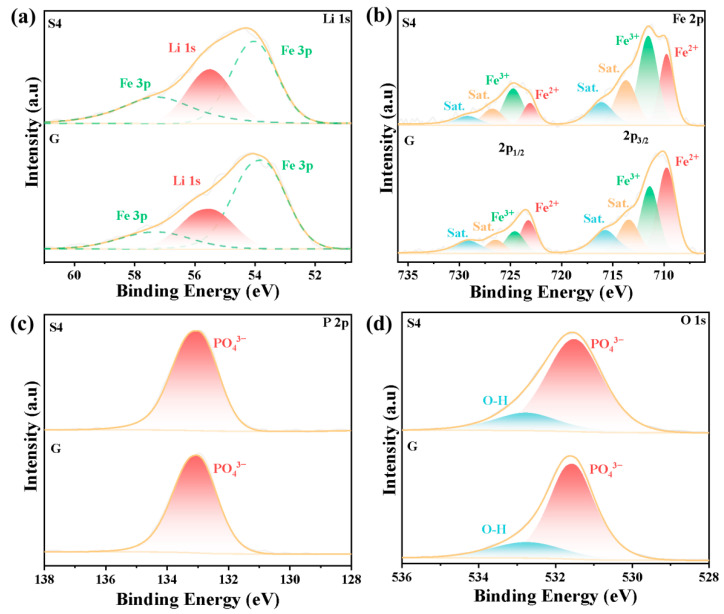
The XPS profiles of samples S4 and G. XPS spectra of (**a**) Li1s, (**b**) Fe2p, (**c**) P2p and (**d**) O1s.

To investigate the valence states and binding energies of the elements in the different samples, XPS tests were conducted on samples S4 and G. The results are illustrated in Figure 6, where the valence states and binding energies of Li, P, and O are consistent with those of previous studies. However, relatively significant differences in the valence states of Fe are observed, as shown in Figure 6b. Split-peak analysis reveals that the fitting results for Li1s, P2p, and O1s of the two samples are largely consistent, with the primary differences observed in the Fe2p region. In LiFePO_4_, Fe^2+^ is characterized by Fe2p2/3 edges near 710.63 eV and 714.47 eV, as well as Fe2p1/2 edges near 724.23 eV and 728.27 eV. These findings indicate that sample S4 contains a greater proportion of Fe^3+^ than sample G does. The elevated Fe^3+^ content in sample S4 may be attributed to its liquid-phase precursor formation, where Fe^2+^ is readily oxidized by atmospheric oxygen to Fe^3+^ in aqueous environments, leading to the observed increase in Fe^3+^ in the XPS spectrum.

### 2.5. Charge–Discharge Performance Analysis

**Figure 7:**
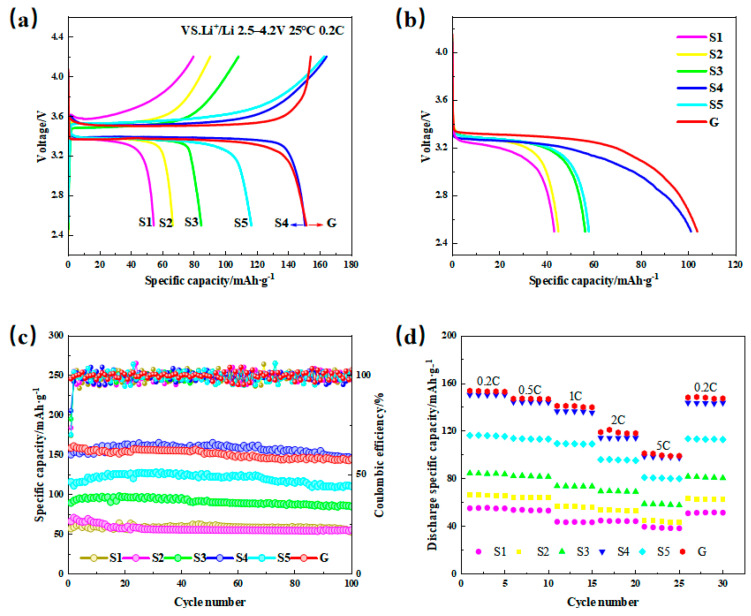
The first charge–discharge curves (**a**) and first discharge curves (**b**) of the different samples at −20 °C. Long cycle stability curves for different samples (**c**) and the rate of multiplication performance curves for different samples (**d**).

The initial charge–discharge performance curves at 25 °C between 2.5 and 4.2 V at a 0.2 C rate are shown in Figure 7a, and all the samples exhibit the charge–discharge curves of typical LFP materials. Overall, the discharge curves of the six materials are relatively smooth, indicating a smooth charging and discharging plateau. The charging platform occurs at 3.5 V, and the discharging platform occurs at 3.4 V, i.e., the platform voltage at which the LiFePO_4_ phase and FePO_4_ undergo phase transition [21,22,23]. In terms of the first discharge specific capacity, sample G synthesized by the ball-milling solid-phase method has a first discharge capacity of 153 mAh·g^−1^, whereas for the sample synthesized by the all-element liquid-phase method, its discharge is highly dependent on the oxalic acid concentration of the antecedent solution.

When the concentration of oxalic acid was 0 mol L^−1^, 0.05 mol L^−1^, 0.1 mol L^−1^, 0.125 mol L^−1^, 0.15 mol L^−1^, the corresponding specific capacities at the first discharge were 55.4 mAh·g^−1^, 68.2 mAh·g^−1^, 86.4 mAh·g^−1^, 150.3 mAh·g^−1^, and 118.7 mAh·g^−1^, respectively. The initial discharge specific capacity of the samples tends to increase but then decreases with increasing oxalic acid concentration. This phenomenon may be attributed to the fact that in an oxalic acid environment with different concentrations, the precursors are produced with different compositional biases, which leads to the Li, Fe, and P in the final material not being in the standard ratios and thus results in capacity differences in the different samples.

The discharge performance of different materials at −20 °C is shown in Figure 7b. The capacity retention rates of samples S1, S2, S3, S4, S5, and G are 79.3%, 67.9%, 66.4%, 70%, 49.7%, and 67%, respectively. All the samples, except for S1 with the smallest capacity, experienced significant capacity decay at low temperatures. Compared with those at room temperature, all the samples except S1 with the lowest capacity exhibited increased capacity attenuation under low-temperature conditions, with their plateau voltages reduced by approximately 0.1 V. In summary, sample S4 demonstrates better capacity retention at low temperatures, with both the voltage attenuation and the magnitude of capacity decay being relatively small, which makes its low-temperature stability more favorable overall.

The long-term cycling stability and corresponding coulombic efficiency of different samples at a charging/discharging rate of 0.2 C are shown in Figure 7c. The first coulombic efficiency of sample G is 90%, whereas those of samples S1, S2, S3, S4, and S5 are 76%, 78%, 81%, 88%, and 80%, respectively. These results indicate that the first coulombic efficiency of the samples synthesized via the all-element oxalic acid liquid-phase method is significantly lower than that of the sample synthesized via the solid-phase method. This discrepancy may be attributed to the liquid-phase synthesis process, where the precursor exhibits compositional biases, resulting in structural defects. These defects impede the reinsertion of partially detaching Li^+^ ions into the crystal lattice after the first charging step, thereby lowering the initial coulombic efficiency.

Furthermore, as shown in Figure 7c, samples S1, S2, S3, S4, S5, and G exhibited capacity retention rates of 92.6%, 91%, 94%, 97.8%, 95.6%, and 90.7%, respectively, after 100 constant-current cycles. Notably, the cycling stability of the samples synthesized by the all-element oxalic acid liquid-phase method surpasses that of the sample synthesized by the solid-phase method following the activation period. These findings align with those of the surface morphology analysis, which highlights that a more homogeneous particle size distribution enhances long-term cycling stability.

The rate performance of the materials is shown in Figure 7d, revealing that the capacity retention rate of LFP synthesized by the all-element oxalic acid-integrated liquid-phase method outperforms that synthesized by the solid-phase method at high rates. Among the samples, S4, characterized by the least attenuation, maintained a capacity of 98.7 mAh·g^−1^ at a rate of 5 C. Its attenuation was notably lower than that of sample G, which retained a capacity of approximately 90.7% at the same rate. This phenomenon can be attributed to the smaller and more uniform particle size of S4, which facilitates faster and more stable Li^+^ insertion and processes during high-rate charging and discharging. Additionally, its relatively stable diffusion performance ensures sustained electrochemical performance across varying rates.

### 2.6. Cyclic Voltammetry Curve Analysis

**Figure 8:**
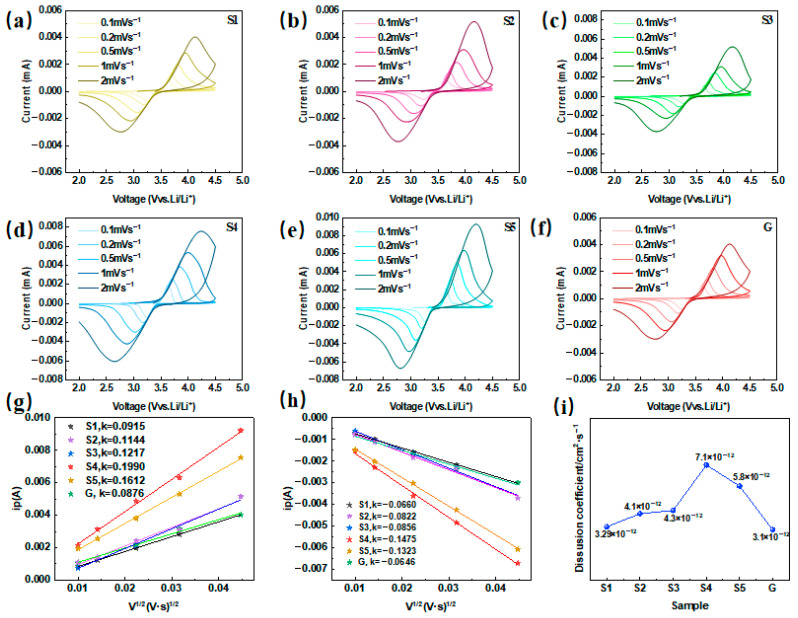
CV curves of samples S1–S5 (**a**–**e**) and G (**f**) at different scan speeds; the relationships between the peak current (ip) of samples S1–S5 and G (**g**,**h**), the sweep speed opening number (V^1/2^) and the diffusion coefficient of different samples (**i**).

The CV curves of samples S1–S5 and G at different scan rates are shown in Figure 8a–f, respectively. These curves reveal that both sets of samples exhibit two distinct peaks at varying scan speeds, corresponding to the redox reactions of LiFePO_4_ during charging and discharging. At a scan rate of 0.1 mVs^−1^, the redox potentials are 3.6 V for S1–S5 and 3.4 V for G. When the scanning voltage reaches 3.6 V, the oxidation reaction occurs, which aligns with the charging and delithiation process of the battery material (Fe^2+^ oxidation to Fe^3+^) [24,25]. Conversely, at 3.4 V, the material undergoes a reduction reaction, corresponding to the lithiation process (Fe^3+^ reduction to Fe^2+^). Furthermore, the oxidation and reduction peaks exhibit complete symmetry, indicating that the electrochemical reactions of the material are stable and reversible. The difference in voltage (ΔV) between the peak voltage and equilibrium potential for the two materials is summarized in Table 3. The data reveal that as the scan rate increases, ΔV also increases, suggesting that the reaction becomes less favorable, which indicates that the electrochemical reactivity of the materials is limited at higher scan rates. A comparison of the data in Figure 8d,f reveals that the redox peak in Figure 8d is more pronounced, demonstrating that the redox reaction of sample S4 is more complete than that of G. This observation highlights the superior electrochemical performance of S4, which is likely due to enhanced lithium-ion mobility and reaction kinetics. To further illustrate the effects of different preparation methods on Li^+^ mobility in materials, the Randle–Sevcik equation was used [26,27,28].

Ip = 2.6 × 10^5^n^3/2^ ACD^1/2^v^1/2^

In the above equation, A is the electrode area, n is the number of electrons per reaction species, D (cm^2^ s^−1^) is the diffusion coefficient of Li^+^, and C is the bulk concentration of Li^+^. After calculation, the lithium ion diffusion coefficients of all the samples are shown in Figure 8i, demonstrating that the lithium ion diffusion coefficients of all the samples are on the same order of magnitude, but the lithium ion diffusion coefficients of the samples synthesized by the all-element integrated liquid-phase method are greater than those of the solid-phase method, and with increasing oxalic acid concentration, the diffusion coefficients increase and then decrease. The maximum value under this method was reached at an oxalic acid concentration of 0.125 mol L^−1^, and this value of 7.1 × 10^−12^ cm^2^ s^−1^ was 2.5 times higher than that of the solid-phase method.

#### AC Impedance Profiling Analysis

**Figure 9:**
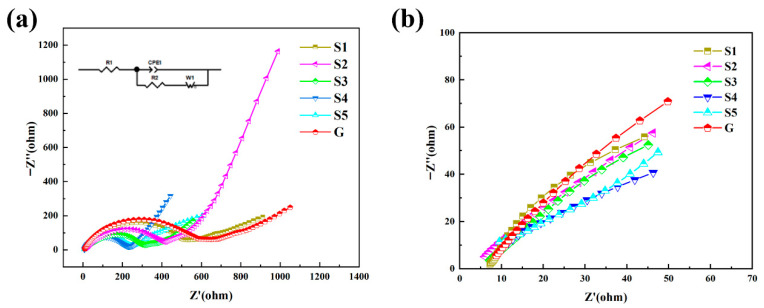
Electrochemical impedance maps and equivalent circuits for different materials (**a**) and EIS impedance mapping between the initial 0–50 Z (ohms) (**b**).

To investigate the impact of different preparation methods on the charge transport properties of the materials, an electrochemical impedance study (EIS) was conducted. The assembled cells were initially activated at 0.2 C and operated within the voltage range of 2.0–4.2 V. Subsequently, an electrochemical impedance test was performed using a Chenhua CHI760E model constant potential instrument operating in a frequency range of 10^–2^ Hz to 10^5^ Hz with a perturbation frequency of 5 mV. The obtained results are illustrated in Figure 9a, with the inset in the upper-left corner depicting the equivalent circuit diagram. Observations from the figure reveal that the impedance data for the two samples consist of a semicircle in the mid- and high-frequency regions and a straight line in the low-frequency region. The semicircles in the mid- and high-frequency regions represent the charge-transfer impedance (Rct) and the conduction impedance (Rs), respectively, which correspond to Li^+^ transport through the solid electrolyte interphase (SEI) membrane. The intercept of the semicircle on the horizontal axis indicates the internal resistance of the system, whereas the straight line in the low-frequency region represents the Warburg impedance (Wo) [29,30]. The impedance curves from 0–50 Ω are presented in Figure 9b. The results demonstrate that the Rct values of samples S1–S5 synthesized under liquid-phase conditions are lower than those of sample G synthesized via the solid-phase method. This suggests that materials developed in liquid-phase environments exhibit superior charge-transfer capabilities. The lower Rct values are attributed to the liquid-phase precursor synthesis, which likely results in a more uniform particle size distribution, lower charge-transfer impedance, and reduced resistance faced by electrons during interface transfer. Consequently, the lower charge-transfer impedance enhances the electrochemical performance of the material. In terms of practical application, this improvement is evident in the faster charging and discharging times and enhanced stability under long cyclic conditions [31,32].

**Figure 10:**
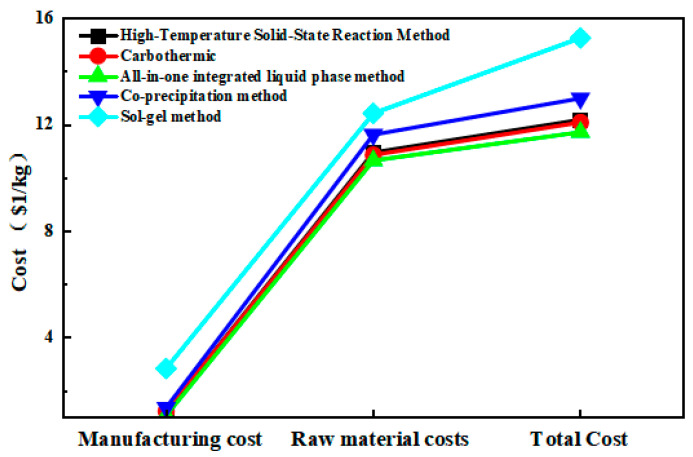
Comprehensive economic comparison of all-in-one integrated liquid phase method with other synthesis methods.

To validate the advantages of the all-in-one integrated liquid phase method over other approaches, we examined its economic benefits, as illustrated in Figure 10. It is evident that the all-in-one integrated liquid phase method synthesis of lithium iron phosphate cathode materials demonstrates considerable advantages in comprehensive aspects such as manufacturing costs, raw material expenses, and energy consumption. Furthermore, Table 4 demonstrates significant differences in raw materials used across various synthesis methods, which also contribute to variations in production costs. In the table, the checkmark symbols represent the commonly used raw materials when synthesizing lithium iron phosphate using the aforementioned method. On the other hand, the cross symbols indicate that such raw materials are not used. Therefore, after comprehensive comparison, the all-in-one integrated liquid phase method holds substantial potential for reducing production costs in lithium iron phosphate synthesis. Detailed data can be found in the Supplementary Material.

## 3. Experimental

### 3.1. Material Preparation

#### 3.1.1. Integrated Liquid-Phase Synthesis of LFP

The iron source used in this experiment was FeC_2_O_4_·2H_2_O (Shanghai Macklin Biochemical Co., Ltd., Shanghai, China), the lithium source was LiOH·H_2_O (Guangdong Yuanfeng Chemical Reagent Co., Ltd., Zhongshan, China), and the phosphorus source was NH_4_H_2_PO_4_ (Tianjin Juhengda Chemical Co., Ltd., Tianjin, China). The specific preparation process is as follows: a series of oxalic acid solutions with varying concentrations were prepared as precursor solutions. The concentrations of oxalic acid were 0 mol L^−1^, 0.05 mol L^−1^, 0.01 mol L^−1^, 0.125 mol L^−1^, and 0.15 mol L^−1^. Li, Fe, and P were weighed in five portions at a molar ratio of 1:1:1 and reserved. The weighed amounts of FeC_2_O_4_·2H_2_O material were added to the respective oxalic acid solutions and stirred thoroughly to obtain homogeneous solutions labeled L1, L2, L3, L4, and L5.

**Figure 11:**
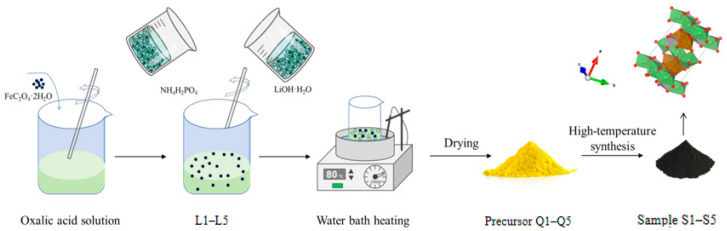
Flow chart of S1–S5 preparation.

Five portions of LiOH·H_2_O and NH_4_H_2_PO_4_ were subsequently prepared at specific concentrations. This solution was slowly and simultaneously added to L1–L5 using a peristaltic pump. Throughout the addition process, continuous stirring was used to guarantee that the reaction proceeded to completion. After the reaction was complete, the reaction vessel was transferred to a water bath and stirred at 80 °C for 1 h. The resulting precursors, labeled Q1, Q2, Q3, Q4, and Q5, were obtained after drying. The dried precursors were ground to a uniform powder and placed into a tube furnace under an argon atmosphere. The furnace was then heated to 740 °C at a rate of 3 °C/min, and the precursors were annealed at this temperature for 12 h to yield a series of LiFePO_4_ cathode materials. Finally, the obtained samples were labeled S1, S2, S3, S4, and S5 after grinding and sieving. The specific preparation process is illustrated in Figure 11.

#### 3.1.2. Synthesis of LFP by the High-Temperature Solid-Phase Method

**Figure 12:**
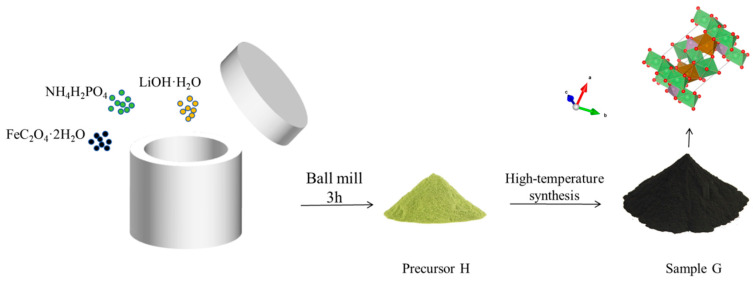
Flow chart of G preparation.

FeC_2_O_4_·2H_2_O, LiOH·H_2_O, and NH_4_H_2_PO_4_ were weighed and combined in a 1:1:1 molar ratio. These weighed reactants were ground in an agate mortar and subsequently transferred into a planetary ball mill jar. An appropriate amount of absolute ethanol was added as a dispersant. Using a JX-4G (Shanghai Jingxin Industrial Development Co., Ltd., Shanghai, China) planetary ball mill, the precursor H was synthesized at a rotational speed of 400 rad min^−1^ for 3 h (with a ball-to-reactor ratio of 4:1). The ground precursor was roasted in a tube furnace equipped with an argon atmosphere, heated to 740 °C at a rate of 3 °C/min, and maintained at that temperature for 12 h. The sample obtained after grinding and sieving was recorded as G. The flow chart for the preparation of G is depicted in Figure 12.

### 3.2. Material Characterization and Chemical Analysis

To determine the crystal structure of the material, a Panaco Empyrean sharp shadow X-ray diffractometer (Panaco, Zoetermeer, The Netherlands) was used for X-ray diffraction (XRD) analysis; the scanning range was 10~90°, and the step size was 0.01° (Cu target Kα radiation λ = 0.154 nm, tube voltage of 40, and tube current of 40 mA). The surface topography of the material was observed using scanning electron microscopy (SEM, SU 8010, Hitachi, Tokyo, Japan) and transmission electron microscopy (TEM, JEM-2100F, JEOL Ltd., Tokyo, Japan). The thermal stability of the materials was characterized using simultaneous thermal analysis (TG–DSC, STA449F3, Netzsch, Selb, Germany). Elemental analysis of the materials was performed using an inductively coupled plasma emission spectrometer (ICP–OES; Agilent 720es, Santa Clara, CA, USA). The materials were characterized by X-ray photoelectron spectroscopy (XPS; Thermo Fisher Scientific, Waltham, MA, USA).

### 3.3. Electrochemical Characteristics

The LIR2032 model of a coin cell was assembled, and the electrochemical properties of the material were tested. The anode active substance, conductive graphite, acetylene black and PVDF were mixed at a ratio of 8:1:1, appropriate amounts of 1-methyl-2-pyroxidone (NMP) were added as the solvent, and the mixed slurry was placed on a magnetic stirrer and stirred for more than 2 h. After stirring, the cathode slurry was evenly coated on the aluminum foil, dried for more than 8 h, and cut into appropriate sizes with a slicer. The subsequent battery assembly occurred in a glove box with a water oxygen content of less than 0.1 ppm. After the assembly, charge/discharge and cycling performance tests were carried out in the voltage range from 2.5 to 4.2 V on a Land Test System (CT2001A, Wuhan Land Electronics Co., Ltd., Wuhan, China). An electrochemical workstation (Chenhua CHl760E, Shanghai Chenhua International Trade Co., Ltd., Shanghai, China) was used for the cyclic voltammetry test and AC impedance test.

## 4. Conclusions

In summary, LiFePO_4_ material was successfully synthesized via the all-element oxalic acid precursor method. This method produces particles with smaller sizes and a more uniform distribution, leading to a greater lithium-ion migration rate and enhanced electrochemical properties. In this method, the particle size of the LiFePO_4_ material can be controlled as needed. Compared with traditional solid-phase synthesis, the all-element oxalic acid precursor method offers several advantages. The precursor is synthesized under ambient conditions (room temperature and pressure) without the need for extensive mixing. This approach reduces energy consumption and lowers the overall preparation cost. Consequently, the all-element oxalic acid precursor method can be used to successfully synthesize LiFePO_4_ material with optimized electrochemical properties. The initial specific discharge capacity of a typical sample prepared is 150.3 mAh·g^−1^, with an initial coulombic efficiency of 88%. The material maintains a high capacity of 98 mAh·g^−1^ even at −20 °C and achieves a discharge capacity of 98.7 mAh·g^−1^ at a high discharge rate of 5 C.

## Figures and Tables

**Table 1:** Structural Parameters Obtained from Rietveld Refinement of S4 and G.

Sample	a (Å)	b (Å)	c (Å)	V (Å^2^)	Rwp (%)	Chi^2^
S4	10.32034	6.00414	4.69094	290.673	6.65	1.21
G	10.32495	6.00617	4.69243	290.994	6.76	1.23

**Table 2:** ICP–OES test results for samples S4 and G.

Sample	Li	Fe	P
S4	4.02	32.11	17.62
G	4.30	33.31	18.50

**Table 3:** Potential differences (ΔV, V) between cathodic (Peak 1, V) peaks and anodic (Peak 2, V) peaks.

**V (mV/s)**		**S1**			**S2**	
	**Peak 1**	**Peak 2**	**ΔV**	**Peak 1**	**Peak 2**	**ΔV**
0.1 mV/s	3.646	3.227	0.419	3.644	3.252	0.392
0.2 mV/s	3.701	3.173	0.528	3.723	3.192	0.531
0.5 mV/s	3.813	3.075	0.738	3.831	3.076	0.755
1 mV/s	3.949	2.939	1.01	3.951	2.933	1.018
2 mV/s	4.123	2.749	1.374	4.156	2.761	1.395
**V (mV/s)**		**S3**			**S4**	
	**Peak 1**	**Peak 2**	**ΔV**	**Peak 1**	**Peak 2**	**ΔV**
0.1 mV/s	3.666	3.23	0.436	3.608	3.291	0.317
0.2 mV/s	3.733	3.184	0.549	3.676	3.178	0.498
0.5 mV/s	3.856	3.046	0.810	3.791	3.059	0.732
1 mV/s	3.975	2.912	1.063	3.912	2.977	0.935
2 mV/s	4.165	2.755	1.41	4.157	2.716	1.441
**V (mV/s)**		**S5**			**G**	
	**Peak 1**	**Peak 2**	**ΔV**	**Peak 1**	**Peak 2**	**ΔV**
0.1 mV/s	3.623	3.237	0.386	3.620	3.237	0.383
0.2 mV/s	3.713	3.167	0.546	3.707	3.163	0.544
0.5 mV/s	3.852	3.026	0.825	3.832	3.022	0.810
1 mV/s	3.994	2.856	1.138	3.998	2.872	1.126
2 mV/s	4.233	2.630	1.603	4.242	2.638	1.604

**Table 4:** Raw materials used across various synthesis methods.

Raw Material	Synthetic Method				
	High-Temperature Solid-State Reaction Method	Carbothermic Reductive Method	All-in-One Integrated Liquid Phase Method	Co-Precipitation Method	Sol–Gel Method
Li_2_CO_3_	√	√	×	√	√
LiOH·H_2_O	×	×	√	×	×
FePO_4_	√	√	×	×	×
FeC_2_O_4_·2H_2_O	×	×	√	×	×
NH_4_FePO_4_	×	×	×	√	×
FeSO_4_·7H_2_O	×	×	×	×	√
NH_4_H_2_PO_4_	×	×	√	×	×
H_3_PO_4_	×	×	×	×	√

**Note:** “√” indicates usage, “×” indicates non-usage.

## Data Availability

The data presented in this study are available upon request from the corresponding authors.

## Supplementary Materials

The following supporting information can be downloaded at: https://www.mdpi.com/article/10.3390/molecules31091419/s1.

## References

[1] Wang G.X., Yang L., Chen Y., Wang J., Bewlay S., Liu H. (2005). An investigation of polypyrrole-LiFePO_4_ composite cathode materials for lithium-ion batteries. Electrochim. Acta..

[2] Xi X., Chen G., Nie Z., He S., Pi X., Zhu X., Zhu J., Zuo T. (2010). Preparation and performance of LiFePO_4_ and LiFePO_4_/C cathodes by freeze-drying. J. Alloys Compd..

[3] Wang Q., Peng D.C., Chen Y.X., Xia X., Liu H., He Y., Ma Q. (2018). A facile surfactant-assisted self-assembly of LiFePO_4_/graphene composites with improved rate performance for lithiumion batteries. J. Electroanal. Chem..

[4] Li Y., Li J., Cao J. (2022). Study on Preparation and Electrochemical Performance of Lithium Iron Phosphate Cathode Ink. Rare Met. Mater. Eng..

[5] Jiang F., Qu K., Wang M., Chen J., Liu Y., Xu H., Huang Y., Li J., Gao P., Zheng J. (2020). Atomic scale insight into the fundamental mechanism of Mn doped LiFePO_4_. Sustain. Energy Fuels.

[6] Cheng Q., Zhao X., Yang G.Y., Mao L., Liao F., Chen L., He P., Pan D., Chen S. (2021). Recent advances of metal phosphates-based electrodes for high-performance metal ion batteries. Energy Storage Mater..

[7] Wang Y.Z., Cui C., Cheng R.F., Wang J., Wang X. (2023). Tannic Acid-Derived Carbon Coating on LiFePO_4_ Nanocrystals Enables High-Rate Cathode Materials for Lithium-Ion Batteries. ACS Appl. Nano Mater..

[8] Sun K., Luo S.H., Du N.Y., Wei Y., Yan S. (2024). Research progress of lithium manganese iron phosphate cathode materials: From preparation to modification. Electroanalysis.

[9] Cheng Y.C., Zhou L., Cai X.L., Cui X., Wu S., Pan W., Li W. (2022). Effect of High-Energy Ball Milling Mixing Process on Electrical Performance and Synthesis Temperature of LiFePO_4_/C Cathode Material. Integr. Ferroelectr..

[10] Liu S.L., Yan P., Li H., Zhang X., Sun W. (2020). One-step microwave synthesis of micro/nanoscale LiFePO4/Graphene cathode with high performance for lithium-ion batteries. Front. Chem..

[11] Chen H., Xu D., Xu J. (2024). LiFe_0.95_Mn_0.05_PO_4_@C film grown on multilayer graphene as a cathode material for lithium-ion batteries. Ionics.

[12] Li Y., Wang L., Zhang K.Y., Yao Y., Kong L. (2021). Optimized synthesis of LiFePO_4_ cathode material and its reaction mechanism during solvothermal. Adv. Powder Technol..

[13] Yang H.T., Pan Z.W., Wang L., Liu C., Wang Z., Zhang C., Lu W. (2023). Enhancing electrochemical performance of Mn doped LiFePO_4_ cathode materials for lithium-ion batteries. Int. J. Electrochem. Sci..

[14] Li Y., Wang L., Zhang H., Liang F., Yao Y., Zhang K. (2021). Freeze drying under vacuum assisted synthesis of LiFePO_4_@MWCNTs composite with phytic acid as phosphorus source for advanced Li-storage. Vacuum.

[15] Hsieh C.-T., Chen I.-L., Chen W.-Y., Wang J.-P. (2012). Synthesis of iron phosphate powders by chemical precipitation route for high-power lithium iron phosphate cathodes. Electrochimi. Acta..

[16] He L. (2012). Synthesis and Modification Study on LiFePO_4_ Cathode Material. Master’s Thesis.

[17] Zhang T., Lin S., Yu J.G. (2022). Influence Mechanism of Precursor Crystallinity on Electrochemical Performance of LiFePO_4_/C Cathode Material. Ind. Eng. Chem. Res..

[18] Peng Y.Q., Zeng L.G., Dai S., Liu F., Rao X., Zhang Y. (2023). LiFePO_4_/C twin microspheres as cathode materials with enhanced electrochemical performance. RSC Adv..

[19] Yang J.J., Guan N.H., Xu C.X. (2024). The synthesis and modification of LiFePO_4_ lithium-ion battery cathodes: A mini review. Crystengcomm.

[20] Huang C.C., Wang L., He X.M. (2016). LiFePO_4_ Crystal Growth during Co-precipitation. Int. J. Electrochem. Sci..

[21] Zhang T., Gong D.G., Lin S., Yu J. (2022). Effect of pH-dependent intermediate on the performance of LiFePO_4_/C cathode material. Chem. Eng. J..

[22] Cheng X.B., Zhang R.Z., Zhao C.Z., Wei F., Zhang J., Zhang Q. (2016). A Review of Solid Electrolyte Interphases on Lithium Metal Anode. Adv. Sci..

[23] Wang A.P., Kadam S., Li H., Shi S., Qi Y. (2018). Review on modeling of the anode solid electrolyte interphase (SEI) for lithium-ion batteries. Npj Comput. Mater..

[24] Wang D.Y., Li H., Shi S.Q., Huang X., Chen L. (2005). Improving the rate performance of LiFePO_4_ by Fe-site doping. Electrochimi. Acta..

[25] Yuan G.H., Bai J.T., Doan T.N.L., Chen P. (2015). Synthesis and electrochemical properties of LiFePO_4_/graphene composite as a novel cathode material for rechargeable hybrid aqueous battery. Mater. Lett..

[26] Morgan D., Vanderven A., Ceder G. (2004). Li conductivity in Li_x_MPO_4_ (M = Mn, Fe, Co, Ni) olivine materials. Electrochem. Solid-State Lett..

[27] Xiao Z.W., Zhang Y.J., Hu G.R. (2015). An investigation into LiFePO_4_/C electrode by medium scan rate cyclic voltammetry. J. Appl. Electrochem..

[28] Yoo Z.W., Zhang K., Patal V. (2018). Porous supraparticles of LiFePO_4_ nanorods with carbon for high-rate Li-ion batteries. Mater. Express..

[29] Zhang Y.T., Xin P.Y., Yao Q.F. (2018). Electrochemical performance of LiFePO_4_/C synthesized by sol-gel method as cathode for aqueous lithium-ion batteries. J. Alloys Compd..

[30] Zhao Q.F., Zhang S.Q., Hu M.-Y., Wang C., Jiang G.-H. (2021). Recent Advances in LiFePO_4_ Cathode Materials for Lithium-Ion Batteries. First-Principles Research. Int. J. Electrochem. Sci..

[31] Lei X.L., Zhang H.Y., Chen Y.M., Wang W., Ye Y., Zheng C., Deng P., Shi Z. (2015). A three-dimensional LiFePO_4_/carbon nanotubes/graphene composite as a cathode material for lithium-ion batteries with superior high-rate performance. J. Alloys Compd..

[32] Jin B., Jin E.M., Park K.-H., Gu H.-B. (2008). Electrochemical properties of LiFePO_4_-multiwalled carbon nanotubes composite cathode materials for lithium polymer battery. Electrochem. Commun..

