# Supplementary material for: Synthesis of Lithium Iron Phosphate Materials via an All-in-One Integrated Liquid Phase Method
Source: Molecules. 2026 Apr 25;31(9):1419. doi: 10.3390/molecules31091419 (PMC13165210; doi:10.3390/molecules31091419)
Supplement: Supplementary file 1 [file molecules-31-01419-s001.zip › molecules-4254734-supplementary.pdf]

Supporting Information

In the Figure 10 and Table 4,we did through all-in-one integrated liquid-phase method along with four other methods High-Temperature Solid-State Reaction Method、Carbothermic reductive method 、Co-precipitation method and Sol-gel method.After conducting an economic comparison of manufacturing costs and raw material costs.(Use the raw material cost at the time of manuscript completion as the reference standard)

Raw material

The price of lithium carbonate is 9.4$/kg.The price of lithium hydroxide monohydrate is 9$/kg.The price of iron phosphate is 1.5$/kg.The price of ferrous oxalate dihydrate is 1.2$/kg.The price of ammonium ferric phosphate is 2.2$/kg.The price of ferrous sulfate heptahydrate is 1$/kg.The price of ammonium dihydrogen phosphate is 0.45$/kg.The price of phosphoric acid is 1.2$/kg.

Manufacturing costs

The high-temperature solid-phase method requires the use of a ball mill for rapid grinding,which is a time-consuming and energy-intensive process.The manufacturing cost is approximately 1.2$/kg.The carbon thermal reduction method is similar to this.The Co-precipitation method requires continuous addition of alkaline solutions throughout the reaction process to strictly control the pH and prevent any deviations in the composition.Therefore,the manufacturing cost is approximately 1.3$/kg.During the synthesis process of the sol-gel method, a large amount of organic solvents are required.Therefore,its manufacturing cost is higher than others,which is approximately 2.8$/kg.So for all-in-one integrated liquid-phase method mentioned in this manuscript, it neither requires high-speed ball milling, nor does it need strict control of pH or the use of organic solvents.Therefore,the manufacturing cost is approximately 1.1$/kg.

Based on the above information, we have mentioned the all-in-one integrated liquid-phase method in this manuscript can save some costs in terms of the raw material and manufacturing.
